# Risk factors and longitudinal changes of dyslipidemia among Chinese people living with HIV receiving antiretroviral therapy

**DOI:** 10.1186/s12879-023-08587-0

**Published:** 2023-09-13

**Authors:** Xiuxia Li, Xiaojing Song, Yang Han, Zhifeng Qiu, Wei Cao, Taisheng Li

**Affiliations:** 1 Department of Infectious Diseases, Peking Union Medical College Hospital, Chinese Academy of Medical Sciences, No.1 Shuaifuyuan, Wangfujing Street, Beijing, 100730 China; 2 Clinical Immunology Center, Chinese Academy of Medical Sciences, Beijing, China; 3 Tsinghua University Medical College, Beijing, China; 4 State Key Laboratory of Complex Severe and Rare Diseases, Peking Union Medical College Hospital, Chinese Academy of Medical Science and Peking Union Medical College, Beijing, China

**Keywords:** HIV infection, Dyslipidemia, Risk factors, Immune activation

## Abstract

**Background:**

Antiretroviral therapy (ART) improved the prognosis of people living with human immunodeficiency virus (HIV) (PLWH). Life-long treatment is required in PLWH and is accompanied by various metabolic abnormalities in the disease course. Data about the epidemiology and the dynamic changes of dyslipidemia in PLWH receiving antiretroviral therapy were scarce in Asian countries. This study aimed to explore the risk factors of dyslipidemia and analyze the longitudinal changes of dyslipidemia among Chinese PLWH receiving HAART.

**Methods:**

We conducted a longitudinal analysis of PLWH enrolled in two large multicenter clinical trials across China, and outpatients followed at the clinic of Peking Union Medical College Hospital. Demographic data and clinical parameters were collected. The risk factors and longitudinal changes in lipid profiles associated with HIV-1 infection were analyzed. The definition of dyslipidemia was made based on the National Cholesterol Education Program, Adult Treatment Panel (NCEP-ATP) III guidelines.

**Results:**

A total of 1542 PLWH were included. The median follow-up was 6 years. At baseline, the concentrations of total cholesterol (TC), triglycerides (TG), high-density lipoprotein cholesterol (HDL-C), and low-density lipoprotein cholesterol (LDL-C) were 4.1 ± 0.91 mmol/L, 1.2 (interquartile ranges [IQR] 0.85–1.75) mmol/L, 1.1 ± 0.37 and 2.4 ± 0.76 mmol/L, respectively. The rate of hypercholesterolemia, hyperglyceridemia, high LDL-C, and low HDL-C were 10.18%, 26.39%, 9.08%, and 44.94%, respectively. The overall prevalence of dyslipidemia was 69.3%, which raised to 84.3% after antiretroviral therapy, substantially higher. CD4/CD8 ratio < 0.3 and viral load > 10^5^ copies/mL were risk factors associated with any subtype of dyslipidemia. A negative correlation between CD8^+^CD38^+^ percentage and HDL-C concentration was found. The regimens including efavirenz (EFV) and tenofovir (TDF) showed better lipid profiles. Longitudinal analysis revealed that both the level and the percentage of abnormal TG and HDL-C occurred drastic change in the first 6 months after ART initiation (from 4.07 to 4.41, from 1.11 to 1.28mmol/L, from 26.39 to 31.1% and from 44.94 to 29.5%, respectively).

**Conclusions:**

The prevalence of dyslipidemia is high in PLWH and increases after ART, mainly represented as high TG and low HDL-C and associated with advanced stage of HIV-1 infection. The greatest changes in lipids occurred in the early stage after initiating ART therapy. The results suggest that dyslipidemia should be monitored and managed when starting ART.

**Supplementary Information:**

The online version contains supplementary material available at 10.1186/s12879-023-08587-0.

## Introduction

Highly active antiretroviral therapy (HAART) has been widely utilized and succeeded in decreasing death related to human immunodeficiency virus (HIV)-associated conditions. Lifelong therapy is required due to the persistent viral reservoir, accompanied by various metabolic complications during longer life [1]. Prior research has shown that dyslipidemia has become a major concern of non-acquired immunodeficiency syndrome (AIDS)-related deaths caused by cardiovascular disease [2, 3]. Compared with the HIV-uninfected populations, the prevalence of overall dyslipidemia is higher [4], ranging from 28 to 80% [5].

Due to the impact of HIV infection on altered lipid metabolism, people living with HIV (PLWH) often display elevated triglycerides (TG) and decreased high-density lipoprotein cholesterol (HDL-C) [6]. Indeed, immunological disorders, the toxicity of antiretroviral drugs, and viral proteins can all contribute to altered lipid metabolism in people living with HIV. The inflammatory response triggered by HIV infection, as well as the toxicity of certain antiretroviral drugs, may impair the efflux of cholesterol from macrophages and modulate the levels of fatty acid synthase [7]. HIV-1-specific viral protein can directly block ATP-binding cassette transporter A1 (ABCA-1)-mediated cholesterol efflux to HDL particles, results in reduced levels of HDL-C [8]. These lipid abnormalities are associated with an increased risk of cardiovascular disease, including heart attacks and strokes [9–11]. Nevertheless, assessment of single indexes cannot describe the lipid metabolism adequately, and complex indexes derived from simple lipid parameters, like non-HDL-C, total cholesterol (TC)/HDL-C, TG/HDL-C, low-density lipoprotein cholesterol (LDL-C)/HDL-C, and non-HDL-C/HDL-C, are widely used in predicting the risk of atherosclerosis in the general population [12–14]. On the other hand, such indexes were scarcely evaluated in PLWH.

Aging, high-calorie diet and higher body mass index are common traditional risk factors for dyslipidemia. In addition, non-traditional hazards, including direct effects on the vasculature of HIV-1 specific viral protein, immune activation, and apparent toxicity from HAART [15], should be mentioned in PLWH. Furthermore, the incidence and severity of dyslipidemia vary based on the types of drugs [16]. The available studies that focused on the prevalence and risk assessment of lipid metabolism disorders among PLWH were mostly carried out in European populations [5], while research in undeveloped countries is limited either by insufficient follow-up or sample size (n = 63, 6-month follow-up [17]; no follow-up [18]; n = 353, no follow-up [19]). In addition, conclusions were inconsistent across studies due to differences in antiretroviral drugs and study populations. For instance, drugs with potential metabolic toxicity are still used broadly in resource-limited countries due to their certain antiviral effectiveness and low price.

Further in-depth studies with a large sample and long duration are warranted to characterize the unique ART-associated lipid profiles and the clinical relevance of these lipid alterations in China. The primary objective of this study was to explore the risk factors of dyslipidemia. The secondary objective was to analyze the longitudinal changes of dyslipidemia among Chinese PLWH receiving HAART.

## Methods

### Study design and participants

This retrospective longitudinal analysis collected data from PLWH originally enrolled in two large multicenter clinical trials across 11 provinces in China and PLWH attending the clinic of infectious disease at Peking Union Medical College Hospital. The subjects included had a high representativeness of the overall population of Chinese PLWH. The details of the clinical trials were described before [20]: Cohort-2009 (CACT0810, ClinicalTrials.gov, identifier NCT00872417) and Cohort-2012 (CACT1215, ClinicalTrials.gov, identifier NCT01844297). The inclusion criteria were confirmed diagnosis of HIV-1 infection, ART-naïve at inclusion, and lipid level data for at least one time point. In contrast, participants with dyslipidemia due to non-AIDS diseases, taking lipid-lowering drugs, suspected adverse drug reactions of non-antiretroviral drugs, or patients without baseline lipid data were excluded.

### Data collection

All subjects were instructed to fast overnight for 12 h before collection of fasting blood samples, preparing for screening semiannually. Plasma lipid levels were measured using an automated chemistry analyzer at the clinical biochemistry laboratories at each study site. T lymphocytes were determined by flow cytometry (FACS Canto, BD Biosciences, NJ, USA) using commercially available monoclonal antibodies, and plasma HIV-1 RNA load was measured using a COBAS Ampliprep/TaqMan 48 Realtime RT-PCR Test (Roche, CA, USA) according to the manufacturer’s instructions. All the laboratories completed a standardization and certification program.

Data were gathered, including complete data with respect to dyslipidemia risk factors. The sociodemographic data, including sex, age, weight, complications like hypertension, type 2 diabetes mellitus (T2DM), opportunistic infection (OI), HIV clinical status, and medication history, were collected by trained study staff at baseline and recorded in electronic research databases. Clinical parameters like TC, LDL-C, HDL-C, TG, CD4^+^ T lymphocyte, CD8^+^ T lymphocyte, CD8^+^CD38^+^%, CD8^+^DR^+^%, and HIV-1 viral RNA were also incorporated.

### Diagnostic criteria

The different subtypes of dyslipidemia were defined as total TC ≥ 5.2 mmol/L, TG ≥ 1.7 mmol/L, HDL-C < 1.04 mmol/L, LDL-C ≥ 3.37 mmol/L, non-HDL-C ≥ 4.1, TC/HDL-C > 5, log (TG/HDL-C) > 0.1, LDL-C/HDL-C > 3.3, or non-HDL-C/HDL-C > 4, based upon the United States National Cholesterol Education Program, Adult Treatment Panel (NCEP-ATP) III guidelines [21].

T2DM was defined as fasting plasma glucose > 7 mmol/L or HbA1c ≥ 6.5% [22]. OIs included the occurrence of infections with *Toxoplasma gondii*, *Pneumocystis jirovecii* (previously *Pneumocystis carinii*), *Cryptococcus neoformans*, *Mycobacterium avium*, *Mycobacterium tuberculosis*, cytomegalovirus, herpes simplex viruses, or *Histoplasma capsulatum*, among others [23]. Hypertension was defined as a systolic blood pressure > 140 mmHg or a diastolic blood pressure > 90 mmHg [24].

### Statistical analysis

All statistical analyses were performed using the SPSS 23.0 statistical software package (IBM Corporation, Armonk, NY, USA). Descriptive data were reported using means, standard deviations, medians, interquartile ranges (IQR), and frequencies. Stratified analyses were assessed using Student’s *t*-test for parametric continuous variables, the Wilcoxon rank-sum test for non-parametric continuous variables, and the chi-square test for categorical variables. Univariable and multivariable logistic regression analyses were performed to assess the risk factors of dyslipidemia using baseline characteristics. The longitudinal data were used to observe the changes of lipid profiles. Variables showing an association in the univariable analyses were evaluated in the multivariable model. All tests were two-tailed, with *P* < 0.05 considered statistically significant.

## Results

### Baseline sociodemographic and clinical characteristics

**Fig. 1:**
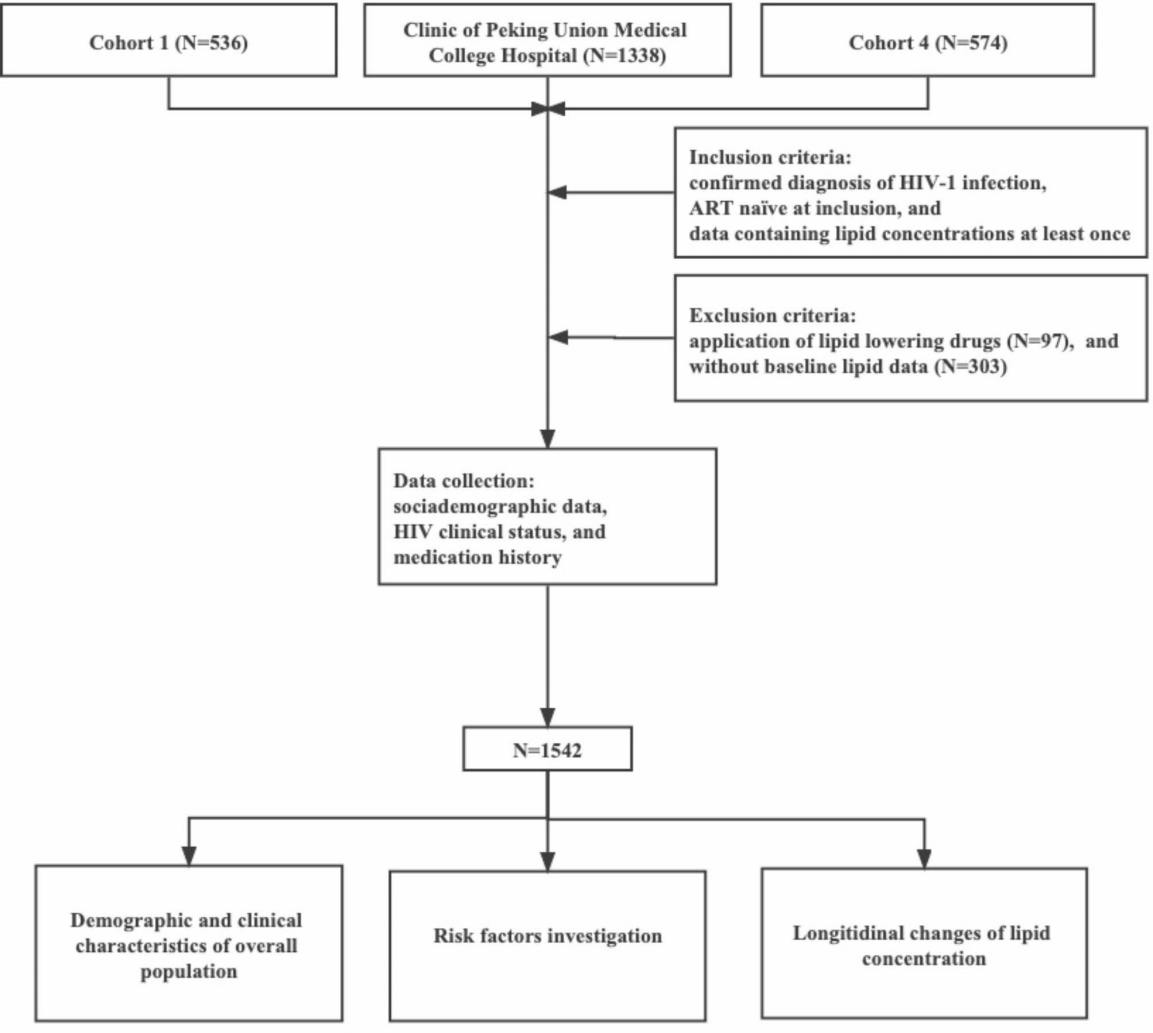
Flow chart of individuals through the screening process

A total of 1542 ART-naïve PLWH were included (Fig. 1). The median follow-up was 6 years (IQR 2.5–7.5), 80.4% (n = 1240) were male, and the mean age was 35.8 ± 10.4 years old. The average body weight was 64.2 ± 11.2 kg. At baseline, 75 PLWH were found to have hypertension, 24 had T2DM, and 293 PLWH were smoking (Table 1).

**Table 1:** Baseline sociodemographic and clinical characteristics

Baseline character	N
Total	1542
Sex	
Male	1240 (80.4%)
Female	298 (19.6%)
Age (years)	
18–29	493 (31.97%)
30–39	496 (32.17%)
40–49	314 (20.36%)
≥ 50	161 (10.44%)
BMI (kg/m^2^)	
≤ 18.5	156 (10.12%)
18.5–24	568 (36.84%)
≥ 24	267 (17.32%)
Hypertension	75 (4.86%)
T2DM	24 (1.56%)
Smoking	293 (19.00%)
OI	202 (13.1%)
CD4 cell count (cells/µL)	
< 200	558 (36.19%)
≥ 200	982 (63.81%)
CD4/CD8 Ratio	
< 0.3	847 (54.93%)
0.3–0.59	500 (32.43%)
≥ 0.6	130 (8.43%)
Viral load (copies/mL)	
< 10^5^	865 (56.09%)
≥ 10^5^	433 (28.08%)

***TC***, **total cholesterol;*TG***, **triglycerides;*HDL-C***, **high-density lipoprotein cholesterol;*LDL-C***, **low-density lipoprotein cholesterol;*Non-HDL-C***, **non-high-density lipoprotein cholesterol;*BMI***, **body mass index;*T2DM***, **type 2 diabetes mellitus;*OI***, Opportunistic infection.*P < 0.05, **P < 0.001

CD4^+^ cell counts lower than 200 cells/µL represented the highest proportion of the PLWH (36.1%, n = 557), and the mean level was 254 (IQR 145–346) cells/µL. The median ratio of CD4/CD8 was 0.3 (IQR 0.16, 0.41), CD8^+^DR^+^% was 65% (54.1-75.3%), and CD8^+^CD38^+^% was 77.1% (64.8-87.6%). Moreover, the mean level of log plasma HIV RNA was 4.7 ± 0.73 copies/mL, with 28.1% (n = 433) of participants higher than 10^5^ copies/mL. Among them, tenofovir (TDF) + lamivudine (3TC) + efavirenz (EFV) accounted for 50.1% (773/1542) of the included PLWH, followed by zidovudine (AZT) + 3TC + nevirapine (NVP) (24.8%, 382/1542), apart from other regimens contained two non-nucleoside reverse transcriptase inhibitors (NNRTIs) + lopinavir/ritonavir (LPV/r) and two NNRTIs + integrase inhibitors (INIs).

### Baseline blood lipid concentrations in PLWH with different clinical characteristics

TC, TG, HDL-C, and LDL-C at baseline were 4.1 ± 0.91 mmol/L, 1.2 (IQR 0.85–1.75) mmol/L, 1.1 ± 0.37 and 2.4 ± 0.76 mmol/L, respectively. Male PLWH had higher levels of TC/HDL-C (*P* < 0.001), LDL-C/HDL-C (*P* < 0.001), non-HDL-C/HDL-C (*P* < 0.001), and decreased HDL-C (*P* < 0.001), while elevated TC (*P* < 0.001) was observed more frequently in female PLWH. Levels of TC (*P* < 0.001), TG (*P* < 0.001), LDL-C (*P* < 0.001), TC/HDL-C (*P* = 0.049), log (TG/HDL-C) (*P* < 0.001), non-HDL-C (*P* < 0.001) were significantly higher in PLWH over 50 years old (*P* < 0.001). Elevation in TC (*P* = 0.012), TG (*P* = 0.002), non-HDL-C (*P* = 0.004), and log (TG/HDL-C) (*P* = 0.04) were found in PLWH with higher body mass index (BMI) (Table 2). PLWH with hypertension has higher TG (*P* = 0.003), TC/HDL-C (*P* < 0.001), non-HDL-C (*P* = 0.016), LDL-C/HDL-C (*P* < 0.001), non-HDL-C/LDL-C (*P* < 0.001) and lower HDL-C (*P* < 0.001). PLWH with OI had higher levels of TG (*P* < 0.001), TC/HDL-C (*P* = 0.003), LDL-C/HDL-C (*P* = 0.009), and non-HDL-C/LDL-C (*P* = 0.008). As for immunological parameters, lower TC, LDL-C, HDL-C, and non-HDL-C, together with higher TG, TC/HDL-C, and log (TG/HDL-C), were found in PLWH with CD4^+^ T lymphocyte < 200/µL. PLWH with CD4/CD8 ratio < 0.3 mainly manifested as significantly lower TC (*P* < 0.01), HDL-C (*P* < 0.01), non-HDL-C (*P* = 0.046), and higher TG (*P* < 0.01), TC/HDL-C (*P* < 0.01), log (TG/HDL-C) (*P* < 0.01), LDL-C/HDL-C (*P* = 0.012), and non-HDL-C/HDL-C (*P* = 0.07). PLWH with viral load over 10^5^ copies/mL brought in higher TG (*P* < 0.001), non-HDL-C (*P* = 0.045), TC/HDL-C (*P* < 0.001), LDL-C/HDL-C (*P* < 0.001), and non-HDL-C/HDL-C (*P* < 0.001), and lower HDL-C (*P* < 0.001).

**Table 2:** Baseline lipid concentrations and Atherosclerotic indexes with different clinical characteristics

Baseline	TC	TG	HDL-C	LDL-C	Non-HDL-C (mmol/L)	TC/HDL-C	lg (TG/HDL-C)	LDL-C/HDL-C	Non-HDL-C/HDL-C
	(mmol/L)	(mmol/L)	(mmol/L)	(mmol/L)					
	Mean ± SD	Median (IQR)	Mean ± SD	Mean ± SD	Mean ± SD	Median (IQR)	Mean ± SD	Mean ± SD	Mean ± SD
Total	4.07 ± 0.91	1.19 (0.85–1.75)	1.12 ± 0.37	2.38 ± 0.76	2.95 ± 0.8	3.73 (3.07–4.48)	0.07 ± 0.3	2.3 ± 0.93	2.89 ± 1.23
Sex									
Male	4.01 ± 0.84 **	1.19 (0.86–1.75)	1.08 ± 0.35 **	2.39 ± 0.73	2.94 ± 0.78	3.79 (3.15–4.57)	0.08 ± 0.3	2.37 ± 0.93	2.97 ± 1.27
Female	4.3 ± 1.1	1.2 (0.83–1.75)	1.28 ± 0.39	2.39 ± 0.86	3 ± 0.88	3.45 (2.86–4.03) **	0.003 ± 0.3	2 ± 0.86 **	2.54 ± 0.97 **
Age (years)									
18–29	3.94 ± 0.77	1.06 (0.78–1.49)	1.13 ± 0.35	2.32 ± 0.73	2.82 ± 0.72	3.6 (2.96–4.29)	0.007 ± 0.29	2.2 ± 0.9	2.76 ± 1.3
30–39	4.09 ± 0.98	1.23 (0.88–1.83)	1.13 ± 0.4	2.4 ± 0.8	2.96 ± 0.82	3.7 (3.03–4.47)	0.08 ± 0.3	2.3 ± 0.99	2.89 ± 1.25
40–49	4.1 ± 0.91	1.29 (0.88–1.81)	1.11 ± 0.35	2.4 ± 0.72	2.99 ± 0.79	3.8 (3.15–4.5)	0.092 ± 0.31	2.33 ± 0.9	2.93 ± 1.16
≥ 50	4.28 ± 1.03 **	1.38 (0.97–2.15) **	1.13 ± 0.37	2.5 ± 0.83 **	3.16 ± 0.93 **	3.99 (3.22–4.6) *	0.13 ± 0.28 **	2.35 ± 0.86	3.02 ± 1.07*
BMI (kg/m^2^)									
≤ 18.5	3.97 ± 0.9	1.14 (0.79–1.63)	1.18 ± 0.33	2.26 ± 0.82	2.79 ± 0.81	3.35 (2.85–4.09)	0.017 ± 0.31	2.04 ± 0.99	2.57 ± 1.21
18.5–24	4.03 ± 0.98	1.22 (0.84–1.75)	1.18 ± 0.35	2.34 ± 0.78	2.85 ± 0.83	3.47 (2.92–4.17)	0.054 ± 0.3	2.13 ± 0.84	2.65 ± 1.13
≥ 24	4.22 ± 0.87*	1.4 (0.96–1.98) **	1.21 ± 0.49	2.42 ± 0.85	3.03 ± 0.77 **	3.68 (2.91–4.27)	0.09 ± 0.31 *	2.18 ± 1.02	2.77 ± 1.19
Hypertension									
Yes	4.14 ± 0.81	1.49 (1.04–1.97)	0.97 ± 0.26	2.48 ± 0.72	3.17 ± 0.75 *	4.29 (3.74–5.13) **	0.21 ± 0.28	2.7 ± 0.99 **	3.47 ± 1.15 **
No	4.06 ± 0.91	1.199 (0.85–1.73) **	1.1 ± 0.37 **	2.38 ± 0.76	2.94 ± 0.8	3.7 (3.04–4.44)	0.06 ± 0.3	2.27 ± 0.92	2.86 ± 1.23
OI									
Yes	4.13 ± 0.96	1.45 (0.94–2.07) **	1.07 ± 0.35	2.48 ± 0.8	3.06 ± 0.86	3.98 (3.18–4.68)	0.14 ± 0.29	2.47 ± 1.02	3.15 ± 1.52
No	4.06 ± 0.9	1.18 (0.84–1.69)	1.13 ± 0.37	2.37 ± 0.75	2.93 ± 0.79	3.7 (3.05–4.43) **	0.06 ± 0.3	2.27 ± 0.91 *	2.85 ± 1.17 *
T2DM									
Yes	4.08 ± 1.06	1.7 (0.91–3.57)	0.89 ± 0.29	2.2 ± 0.67	3.18 ± 0.99	4.9 (3.62–5.45)	0.35 ± 0.41	2.60 ± 0.88	3.86 ± 1.64
No	4.07 ± 0.94	1.2 (0.85–1.74)	1.12 ± 0.37	2.39 ± 0.76	2.94 ± 0.82	3.7 (3.05–4.45)	0.06 ± 0.29	2.29 ± 0.93	2.87 ± 1.22
Smoking									
Yes	4.05 ± 0.82	1.3 (0.92–1.99)*	1.13 ± 0.40	2.4 ± 0.77	2.92 ± 0.81	3.71 (3.03–4.45)	0.11 ± 0.32	2.3 ± 0.96	2.87 ± 1.32
No	4.07 ± 0.93	1.17 (0.84–1.71)	1.12 ± 0.36	2.38 ± 0.76	2.95 ± 0.83	3.73 (3.08–4.49)	0.06 ± 0.29	2.3 ± 0.93	2.89 ± 1.22
CD4 cell count (cells/µL)									
< 200	3.94 ± 0.96 **	1.25 (0.89–1.8) *	1.06 ± 0.33 **	2.31 ± 0.75 *	2.87 ± 0.8 *	3.82 (3.08–4.55) *	0.11 ± 0.29*	2.34 ± 0.93	2.96 ± 1.2
≥200	4.14 ± 0.87	1.16 (0.84–1.71)	1.15 ± 0.38	2.43 ± 0.76	3 ± 0.8	3.69 (3.07–4.43)	0.05 ± 0.29	2.27 ± 0.93	2.85 ± 1.25
CD4/CD8 Ratio									
< 0.3	3.99 ± 0.94 **	1.28 (0.92–1.81) **	1.06 ± 0.32 **	2.35 ± 0.76	2.93 ± 0.82 *	3.85 (3.19–4.58) **	0.11 ± 0.29 **	2.36 ± 0.93 *	2.98 ± 1.21 *
0.3–0.59	4.1 ± 0.85	1.11 (0.8–1.64)	1.17 ± 0.41	2.4 ± 0.78	2.95 ± 0.78	3.61 (2.97–4.44)	0.02 ± 0.3	2.24 ± 0.96	2.79 ± 1.28
≥ 0.6	4.35 ± 0.86	1.05 (0.76–1.84)	1.23 ± 0.38	2.5 ± 0.69	3.11 ± 0.8	3.58 (2.95–4.34)	0.01 ± 0.34	2.17 ± 0.78	2.74 ± 1.07
Viral load (copies/mL)									
< 10^5^	4.1 ± 0.82	1.1 (0.8–1.57)	1.17 ± 0.37	2.4 ± 0.71	2.94 ± 0.75	3.63 (2.99–3.83)	0.01 ± 0.29	2.21 ± 0.83	2.73 ± 1.02
≥ 10^5^	4.07 ± 1.05	1.4 (0.99–1.96) **	1.02 ± 0.34 **	2.33 ± 0.87	3.04 ± 0.88 *	4.06 (3.4–4.85) **	0.17 ± 0.3**	2.47 ± 1.13 **	3.3 ± 1.55 **

***TC***, **total cholesterol;*LDL-C***, **low-density lipoprotein cholesterol**; ***TG***, **triglycerides;*HDL-C***, **high density lipoprotein cholesterol;*non-HDL-C***, **non-high-density lipoprotein cholesterol**; ***SD***, **standard deviation;*BMI***, **body mass index;*OI, o*pportunistic infection;*T2DM***, **type 2 diabetes mellitus;*TDF***, **tenofovir**; ***3TC***, **lamivudine**; ***EFV***, **efavirenz;*AZT***, **zidovudine**; ***NVP***, nevirapine.*P < 0.05, **P < 0.001

### Risk factors associated with dyslipidemia in PLWH (N = 1542)

**Fig. 2:**
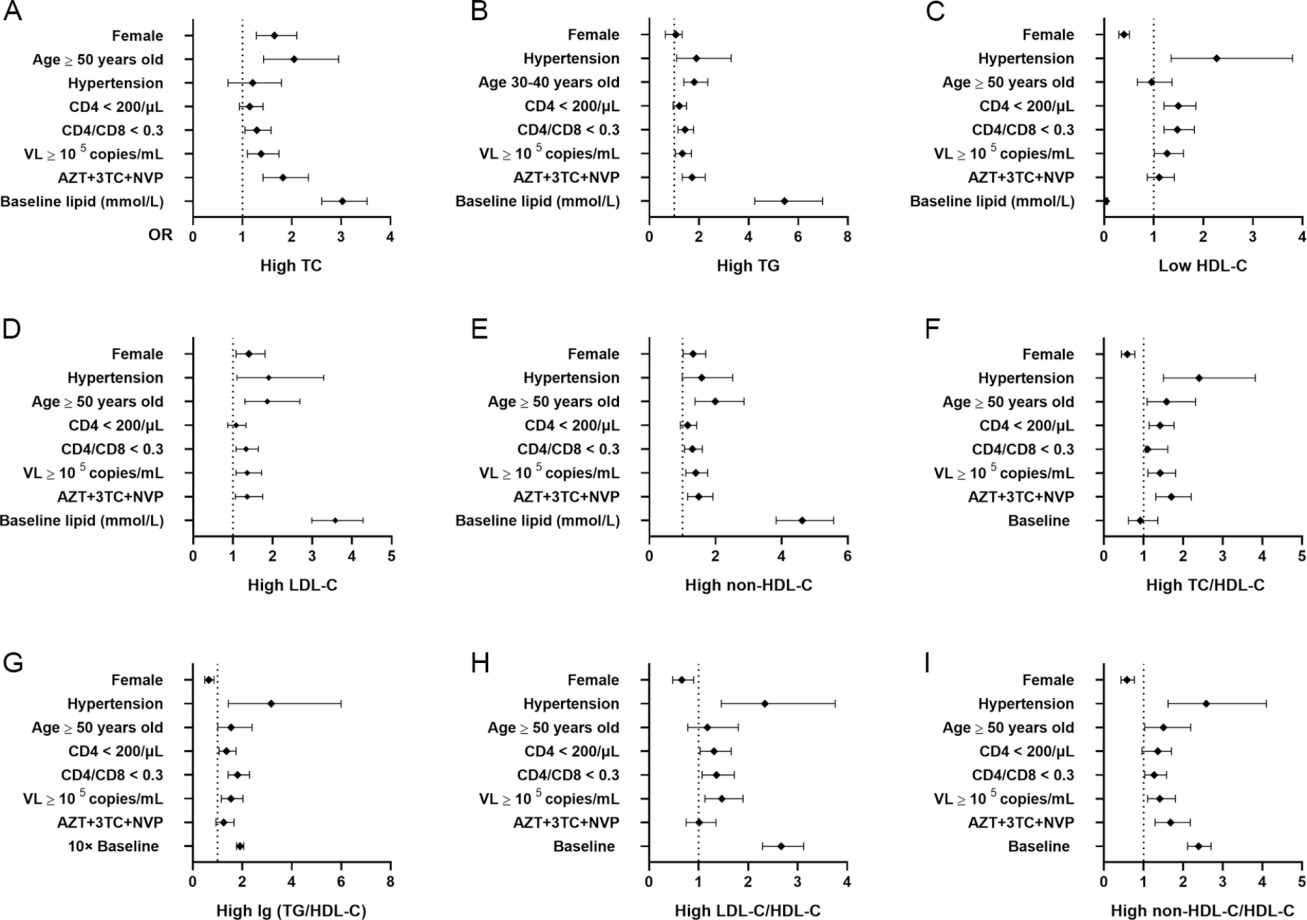
Univariable Binary logistic regression analysis for risk factors of dyslipidemia. The line with filled dots represented the odds ratio and 95% confidential interval. The value of the odds ratio was shown on X-Axis. **A** Risk factors of high TC. **B** Risk factors of high TG. **C** Risk factors of low HDL-C. **D** Risk factors of high LDL-C. **E** Risk factors of high non-HDL-C. **F** Risk factors of high TC/HDL-C. **G** Risk factors of high log (TG/HDL-C). **H** Risk factors of high LDL-C/HDL-C. **I** Risk factors of high non-HDL-C/HDL-C. TC, total cholesterol; TG, triglycerides; HDL-C, high-density lipoprotein cholesterol; LDL-C, low-density lipoprotein cholesterol; AZT, zidovudine; 3TC, lamivudine; NVP, nevirapine

The univariable binary logistic regression analyses showed that female sex, age > 50 years old, HIV-1 RNA over 10^5^ copies/mL, receiving ART regimens containing AZT and CD4/CD8 ratio < 0.3 were shared risk factors for raised TC, LDL-C, and non-HDL-C, which formed the core of atherosclerosis directly. Regarding parameters like TG and HDL-C, abnormal levels were more prevalent in PLWH with hypertension, lower CD4/CD8 ratio, or higher viral load. Concerning atherogenic indexes like TC/HDL-C, log (TG/HDL-C), LDL-C/HDL-C, and non-HDL-C/HDL-C, increased age, complicated with hypertension, decreased CD4/CD8 ratio and HIV-1 RNA more than 10^5^ copies/mL were common risk factors (Fig. 2; Additional File 1, Table S1).

The multivariable binary logistic regression analysis showed that male PLWH were at a higher risk of abnormal HDL-C, TC/HDL-C, log (TG/HDL-C), and non-HDL-C/HDL-C, while female PLWH would develop high TC more easily. In the context of older adult PLWH, high TC, and elevated LDL-C were more frequent. In addition, hypertension was an independent risk factor for high TG and all four abnormal atherogenic indexes. With respect to specific variables associated with HIV-1 infection, decreased ratio of CD4/CD8 and male were independent risk factors for low HDL-C and high log (TG/HDL-C). Besides, HIV RNA > 10^5^ copies/mL was an independent risk factor for high TC, TG, LDL-C, non-HDL-C, TC/HDL-C, LDL-C/HDL-C, and non-HDL/HDL-C. In a similar situation, using a regimen containing 3TC + AZT + NVP was prone to increase the risk of high TC, TG, LDL-C, non-HDL-C, TC/HDL-C, and non-HDL/HDL-C when using regimens consisting of 3TC + TDF + EFV as a reference (Table 3).

**Table 3:** Multivariate Binary logistic regression analysis for risk factors of dyslipidemia

Subtype of dyslipidemia	TC	TG	HDL-C	LDL-C	Non-HDL-C	TC/HDL-C	log (TG/HDL-C)	LDL-C/HDL-C	non-HDL-C/HDL-C
	OR (95%CI)	OR (95%CI)	OR (95%CI)	OR (95%CI)	OR (95%CI)	OR (95%CI)	OR (95%CI)	OR (95%CI)	OR (95%CI)
Male	0.68 (0.51–0.91) *	——	2.52 (1.84–3.44) **	——	——	1.61 (1.14–2.28) *	1.7 (1.24–2.32) *	——	1.99 (1.41–2.8) **
Age ≥ 50 years old	1.79 (1.18–2.72) *	——	——	1.64 (1.09–2.48) *	1.66 (1.1–2.52) *	——	——	——	——
Hypertension	——	2.84 (1.39–5.8) **	——	——	——	2.15 (1.21–3.82) **	3.52 (1.37-9) **	2.16 (1.23–3.8) **	2.2 (1.22–3.87) **
CD4/CD8 < 0.3	——	——	2.4 (1.51–3.81) **	——	——	——	1.71 (1.31–2.25) *	——	——
Viral load ≥ 10 ^5^ copies/mL	1.52 (1.18–1.96) *	1.31 (1.01–1.71) *	——	1.42 (1.1–1.82) *	1.44 (1.12–1.86) *	1.42 (1.09–1.85) *	——	1.39 (1.06–1.84) *	1.3 (1.01–1.7) *
AZT + 3TC +NVP	2.29 (1.69–3.1) **	1.58 (1.15–2.17) *	——	1.5 (1.11–2.02) *	1.84 (1.36–2.48) **	2.13 (1.54–2.95) **	——	——	1.87 (1.37–2.58) **

***TC***, **total cholesterol;*TG***, **triglycerides;*HDL-C***, **high-density lipoprotein cholesterol;*LDL-C***, **low-density lipoprotein cholesterol;*Non-HDL-C***, **non-high-density lipoprotein cholesterol;*AZT***, **zidovudine;*3TC***, **lamivudine;*NVP***, **nevirapine;*OR***, **odds ratio;*CI***, confidence interval. *P < 0.05, **P **< 0.01**

### Preliminary analysis of the relationship between CD8 immune activation and dyslipidemia

**Fig. 3:**
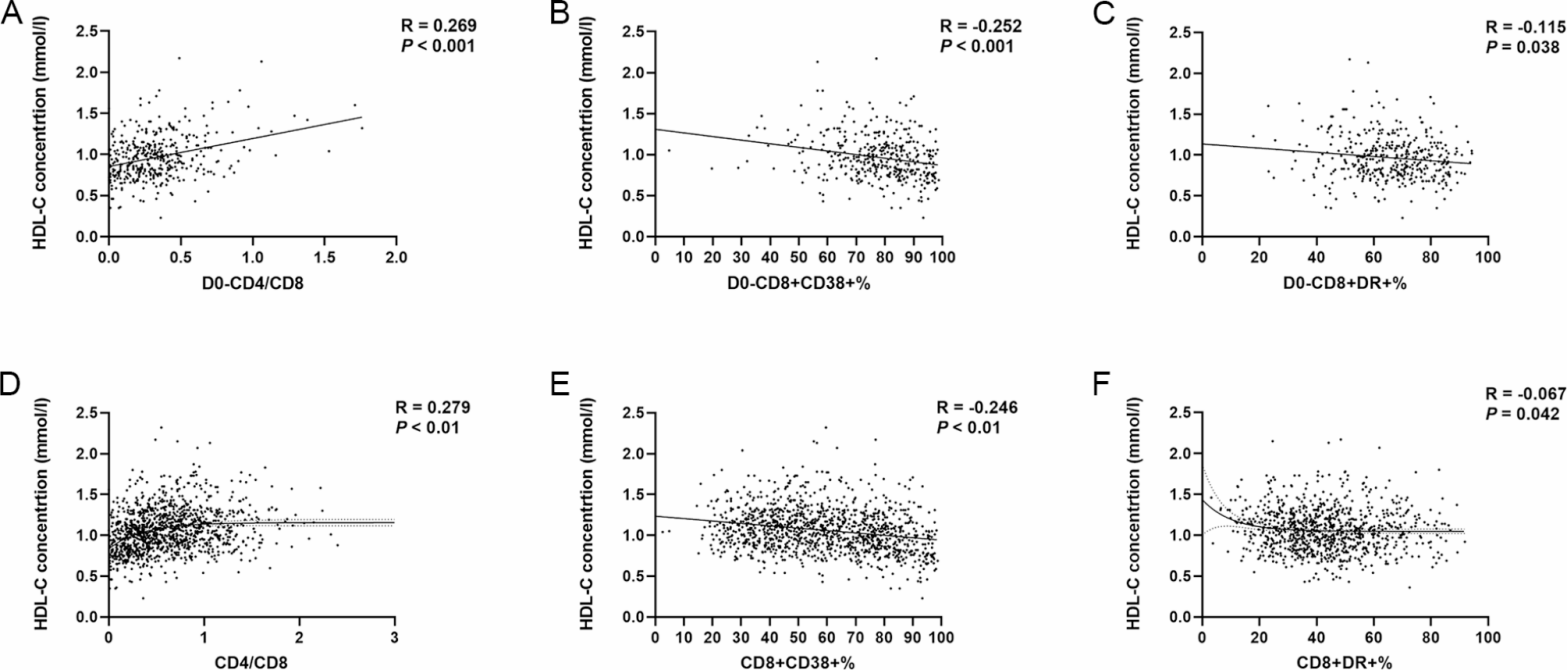
The correlation between markers of immune activation and the concentration of HDL-C. Pearson/Spearman Analysis was conducted. **A** The correlation between D0-CD4/CD8 activation and the concentration of HDL-C. **B** The correlation between D0-CD8 + CD38+% activation and the concentration of HDL-C. **C** The correlation between D0-CD8 + DR+% activation and the concentration of HDL-C. **D** The correlation between CD4/CD8 activation and the concentration of HDL-C. **E** The correlation between CD8 + CD38+% activation and the concentration of HDL-C. **F** The correlation between CD8 + DR+% activation and the concentration of HDL-C. HDL-C, high-density lipoprotein cholesterol; D0, baseline

HDL-C was positively correlated with CD4^+^ T cell count (R = 0.211, *P* < 0.01) and CD4/CD8 ratio (R = 0.279, *P* < 0.01), while inversely related to CD8^+^CD38^+^% (R = -0.246, *P* < 0.01) as well as an extremely weak relationship with CD8^+^DR^+^% during the whole process (R = -0.067, *P* = 0.042). No statistical significance was found in other types of lipids (Fig. 3).

### Longitudinal changes in the lipid concentration and the proportions of dyslipidemia across a 6-year follow-up (N = 1542)

The percentage of hypercholesterolemia, hypertriglyceridemia, low HDL-C, high LDL-C, and the overall rate of dyslipidemia before HAART initiation was 10.18%, 26.39%, 44.94% 9.08%, and 69.3%, respectively. HAART use was generally correlated with higher lipid concentrations. During follow-up, the percentage of dyslipidemia raised to 84.3% after antiretroviral therapy, 51.69% for high TC, 67.26% for high TG, 58.11% for low HDL-C, and 38.44% for high LDL-C. Furthermore, the rate of severe hyperglyceridemia (≥ 5.6 mmol/L) was 12.36% and 2.8% for severe high LDL-C (≥ 4.9 mmol/L).

**Fig. 4:**
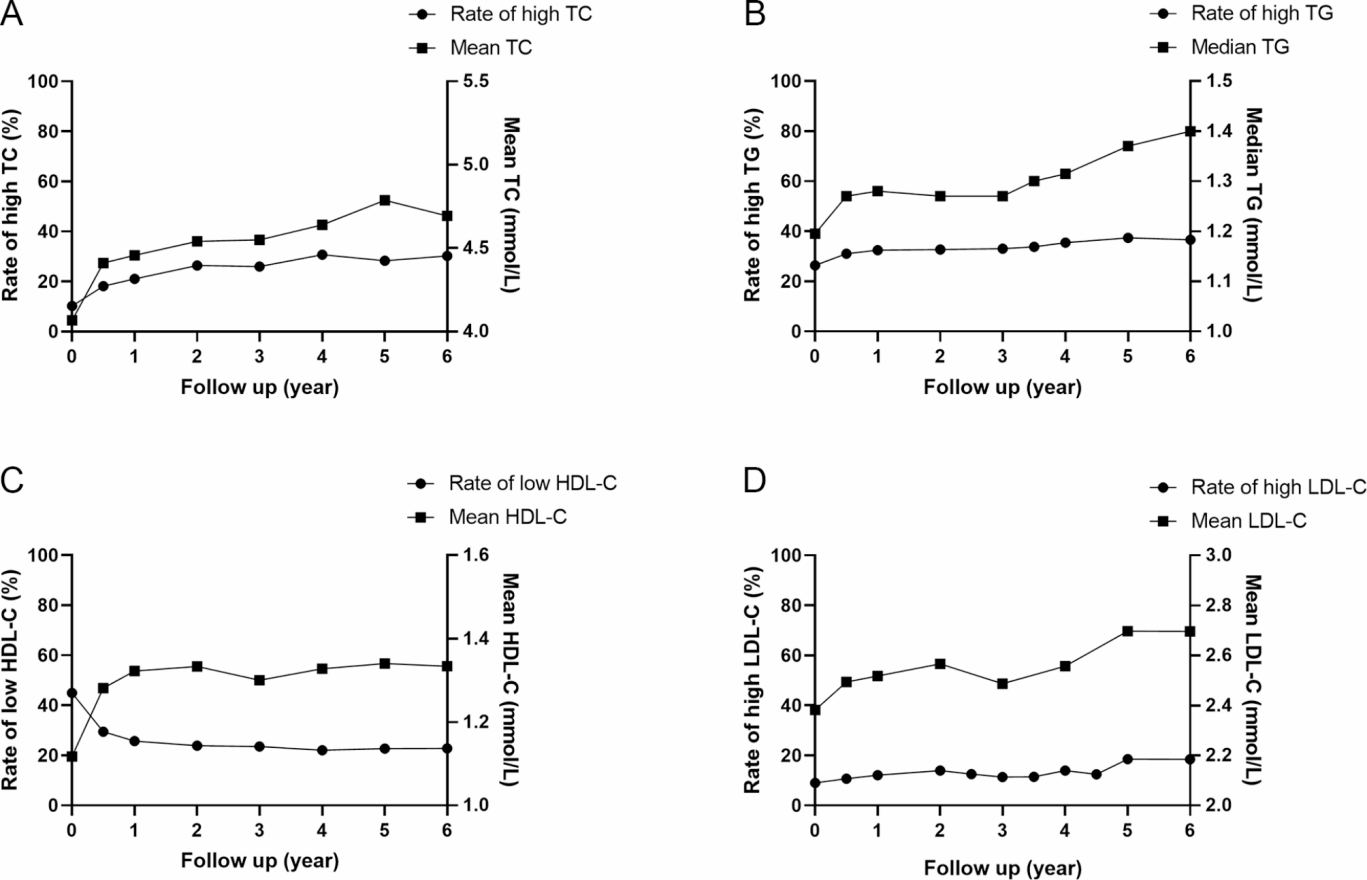
Longitudinal changes of lipid. The line with filled dots represented the proportion of abnormal lipid (left ordinated axis), and the line with solid square indicated the concentration of lipid (right ordinated axis). The number of individuals at various follow-up points was 1542 (100%), 1456 (94.4%), 1330 (86.25%), 1025 (66.47%), 902 (58.49%), 904 (58.6%), and 694 (45%), respectively. **A** Longitudinal change in TC. **B** Longitudinal changes of TG. **C** Longitudinal changes of HDL-C. **D** Longitudinal changes of LDL-C. TC, total cholesterol; TG: triglycerides; HDL-C, high-density lipoprotein cholesterol; LDL-C, low-density lipoprotein cholesterol

Compared with the baseline, the percentage of dyslipidemia changed significantly after ART initiation during the 6-year follow-up. Simultaneously the percentage of hypercholesterolemia (Fig. 4A), hypertriglyceridemia (Fig. 4B), and hyper LDL-C (Fig. 4D) all increased from baseline to the 6th year, with significant differences (10.18% vs. 17.6%, 18.13%, 21.05%, 22.73%, 26.39%, 29.09%, 25.94%; 26.39% vs. 31.10%, 32.33%, 32.71%, 33.07%, 35.48%, 37.35%, 36.6%; 9.08% vs. 10.7%, 12.16%, 13.95%, 11.34%, 13.95%, 18.5%, 18.5%; F = 181.4, 44.83, 66.37, respectively, all *P* < 0.001). On the contrary, the percentage of low HDL-C (Fig. 4C) decreased along with prolonged HAART (44.94% vs. 29.5%, 25.71%, 23.88%, 23.58%, 22.08%, 22.68%, 22.77%; F = 281.5, *P* < 0.001). During HAART, the proportion of high TG was maintained at the highest level, while the percentage of high LDL-C stayed the lowest. Low HDL-C was more common than high TC at the early stage of ART, yet hypercholesterolemia was more commonly seen as time prolonged (see Additional File 2, Fig. S1).

Surprisingly, the incidence of new-onset high TG and low HDL-C decreased over 6 years, with a sharp decline in the first 6 months after ART initiation (from 26.4 to 14.7% and from 44.94 to 6.4%, respectively). In contrast, no remarkable changes were observed in the incidence of elevated TC and LDL-C, which leveled off at 10% over the 6 years of follow-up (see Additional File 3, Fig. S2).

Regarding lipid levels, similar increasing trends were found in the levels of TC (Fig. 4A), TG (Fig. 4B), HDL-C (Fig. 4C), and LDL-C (Fig. 4D); they increased from baseline to varying degrees with the extension of time (4.07 vs. 4.41, 4.46, 4.54, 4.55, 4.64, 4.79, 4.7 mmol/L; 1.19 vs. 1.27, 1.28, 1.27, 1.25, 1.32, 1.37, 1.4 mmol/L; 1.11 vs. 1.28, 1.32, 1.33, 1.30, 1.33, 1.34, 1.33 mmol/L; 2.38 vs. 2.49, 2.52, 2.57, 2.49, 2.55, 2.69, 2.7 mmol/L; F = 68.63, 10.88, 46.93, 14.12, respectively, all *P* < 0.001).

## Discussion

The broad use of HAART has greatly improved HIV-relevant conditions, but the life-long treatment brought in complications like dyslipidemia, which may enhance CVD risk. There are limited data about dyslipidemia in Chinese PLWH. This study described the dynamics of lipid metabolism in PLWH with a median follow-up of 6 years and explored the various risk factors of dyslipidemia from two large multicenter clinical trials in China.

The results showed that the overall prevalence of dyslipidemia was 69.3% upon enrollment, which increased to 84.3% after ART, with hypertriglyceridemia and decline in HDL-C being the most common abnormality, in line with other findings in low- and middle-income countries [17, 25]. In addition, atherogenic indexes consisting of lipid parameters like log (TG/HDL-C) were found to be elevated more obviously. Dyslipidemia associated with HIV-1 infection may accelerate atherosclerosis and coronary disease [26]. The results demonstrated a low rate of CVD or pancreatitis despite the high rate of lipid disturbance, supported by earlier research [27]. In line with the known traditional cardiovascular risk factors in the general population [28, 29], advanced age and hypertension were independent risk factors for most lipid indexes in PLWH.

Depletion of CD4 + T cells and aberrant immune activation are typical features of HIV-1 infection, closely related to non-AIDS-defining disorders. The complex relationship between lipids and HIV-1 infection, as immunological disorders, may be both a cause and a consequence of lipid abnormalities. Inflammation may impair cholesterol efflux from macrophages via reverse cholesterol transport and aggravate the oxidative modification of LDL [7], modulating levels of fatty acid synthase and altering the plasma lipid profiles. Vice versa, oxidative cholesterol accumulation in macrophages promotes inflammatory responses, including augmentation of immune activation, leading to exacerbation of HIV-1 progression [7]. Therefore, the association between lipid parameters and markers of CD4^+^ cell count, CD4/CD8 ratio, CD8^+^CD38^+^%, and CD8^+^DR^+^% were investigated. The findings showed a similar trend to a previous report [9], i.e., higher rates of abnormal TC, HDL-C, TC/HDL-C, and log (TG/HDL-C) were seen among those with CD4^+^ T cells < 200/µL. Still, Pefura Yone et al. [30] reported a trend of ascending prevalence of dyslipidemia with increasing CD4 T cell counts in PLWH under HAART. It might be because HAART leads to partial immune restoration and nutrition enhancement contributing to elevated lipids. In addition, the toxicity of HAART plays a major insidious role in developing poor lipid profiles despite the increasing CD4 count.

Inversion of the CD4/CD8 ratio has been identified as a hallmark of immune senescence and a predictor of early atherosclerosis [31]. Current results identified that the lower the ratio, the higher the risk of lipid disorder would be despite the lipid subtype. The multivariable analysis showed that a CD4/CD8 ratio < 0.3 was an independent risk factor for low HDL-C and high log (TG/HDL-C). A prospective cohort study suggested that positive associations were observed between oxidized lipoproteins and most markers of inflammation [32]. Still, few studies focused on the relevance between immune activation of CD8^+^ T cells and lipid metabolism though the former marker was reported to be associated with subclinical atherosclerosis [33]. In order to address this gap, 398 PLWH with complete data of immune activation markers were analyzed. The results demonstrated a negative correlation between HDL-C concentration and CD8^+^CD38^+^% during a 5-year follow-up, though no significant relationships were found between immune activation markers and other lipid species. These results helped confirm that the dysregulated immune status probably played an important role in HIV-associated dyslipidemia. The present study suggests that it might help to promote the restoration of dyslipidemia by developing strategies for managing immune activation.

Apart from immune disorders, the HIV-1-specific viral protein can directly block ABCA-1-mediated cholesterol efflux to HDL particles, resulting in intracellular accumulation of lipids and enhanced foam cell formation [8]. In accordance with previous research [9–11]. The multivariable analysis showed that a viral load over 10^5^ copies/mL was an independent risk factor for most types of dyslipidemia. Special attention should be paid to PLWH at the progressive stage, and early antiretroviral therapy was advocated to avoid potential dyslipidemia.

HAART provides both benefits and risks for HIV-infected with respect to lipid profile. Older drugs consisting of NNRTIs like EFV and NVP or certain NRTIs like AZT and TDF are key components of the first-line HAART regimens in China due to the limited drug resource, despite that they might not have optimal safety profiles. The specific effects of the above drugs on lipids are not fully described in Chinese research. In this study, HAART affected the rate of high TG and elevated TC more pronouncedly, in accordance with other studies [34, 35]. Among that, AZT co-formulated with NVP had more adverse effects on lipid concentrations than EFV co-formulated with TDF. The multivariable analysis showed that the regimen consisting of AZT, NVP, and 3TC was an independent risk factor for abnormal TC, LDL-C, non-HDL-C, TG, TC/HDL-C, and log (TG/HDL-C). Further studies are warranted to investigate the underlying mechanisms of how ART influences lipid levels and determine how much lipid abnormalities translate into cardiovascular disease risk.

Lipid changes over time were not monotonic. Long-term toxicities become apparent because of cumulative HAART exposure, immunosuppression, viremia, and other individual elements. Therefore, this study analyzed the dynamics of lipid changes during follow-up. Surprisingly, most lipid occurred drastic change in the first 6 months after HAART initiation and minor fluctuation subsequently, contributing to the emphasis on monitoring blood lipids early after HAART application. Among that, it is worth mentioning that the levels of HDL-C returned to normal. This correction in lipid profile is perhaps largely due to the suppression of viral replication and restoration of immune function following ART initiation, representing a return toward a less proinflammatory state.

The strengths of this study include a large sample size of national representation and a relatively long follow-up along longitudinal analyses, contributing to enhancing the trustworthiness of results. In addition, the analysis included comprehensive lipid markers, which are evaluated the most frequently in clinical practice and other studies. In the context of risk factors, we verified the relationship between progressive clinical stage and unfavorable lipid profile alterations from multiple dimensions. In particular, an innovative interplay was found between CD8^+^CD38^+^% and abnormal HDL-C.

There were some limitations that may impact the quality of current research. Firstly, loss to follow-up of blood lipids occurred at different time points, and the data presented are partly missing within the context of a retrospective design. Secondly, expanded information about behavioral changes such as diet and physical activity and family history in clinical practice was not recorded. Thirdly, the longitudinal data only included lipid data, and the changes in T2DM, hypertension, and other cardiovascular risk factors were unavailable, and the association between these changes could not analyzed. Fourthly, only the occurrence of the events was collected during follow-up and not the time of occurrence. Therefore, Cox regression and Kaplan-Meier analyses were not possible. Sixthly, data regarding specific medications like thiazide and β-blockers were not collected. In order to overcome the above shortcomings, prospective well-controlled cohort studies should be performed to assess the detailed risk factors associated with dyslipidemia in Chinese PLWH and uninfected populations, especially inflammation. Besides, advanced techniques to evaluate the detailed lipid profile are imperative to explore the in-depth mechanism of dyslipidemia in PLWH.

## Conclusions

The rate of dyslipidemia, mainly high TG and low HDL-C, was high in PLWH, while few events of cardiovascular disease or pancreatitis were found. Of note, the lipid levels of the aging population or individuals at the advanced stage of HIV-1 infection should be carefully examined. Careful monitoring in the first 6 months after ART initiation and timely treatment of HARRT should be advocated in PLWH due to the complex and multidirectional relationships among ART, viral replication, chronic inflammation, and lipid metabolism.

### Electronic supplementary material

Below is the link to the electronic supplementary material.

Supplementary Material 1

Supplementary Material 2

Supplementary Material 3

## Data Availability

The datasets used and/or analyzed in the current study are available from the corresponding author upon reasonable request.

## Abbreviations

HIV: human immunodeficiency virus
PLWH: people living with HIV
HAART: highly active antiretroviral therapy
TG: triglycerides
HDL-C: high-density lipoprotein cholesterol
TC: total cholesterol
LDL-C: low-density lipoprotein cholesterol
T2DM: type 2 diabetes mellitus
OI: opportunisty infection
NCEP-ATP: National Cholesterol Education Program, Adult Treatment Panel
IQR: interquartile ranges
NNRTIs: non-nucleoside reverse transcriptase inhibitors
NRTI: nucleoside reverse transcriptase inhibitor
PLWH: people living with HIV
TDF: tenofovir
3TC: lamivudine
EFV: efavirenz
AZT: zidovudine
NVP: nevirapine
LPV/r: Lopinavir/ritonavir
INIs: integrase inhibitors
BMI: body mass index
CVD: cardiovascular disease
HTGP: hypertriglyceridemia pancreatitis
LDL: low-density lipoprotein
ARV: antiretroviral virus
HDL: high-density lipoprotein
ABCA-1: ATP-binding cassette transporter A1
