# Supplementary material for: Risk factors and longitudinal changes of dyslipidemia among Chinese people living with HIV receiving antiretroviral therapy
Source: BMC Infect Dis. 2023 Sep 13;23:598. doi: 10.1186/s12879-023-08587-0 (PMC10500758; doi:10.1186/s12879-023-08587-0)
Supplement: Supplementary file 2 — Supplementary Material 2 [file 12879_2023_8587_MOESM2_ESM.pdf]

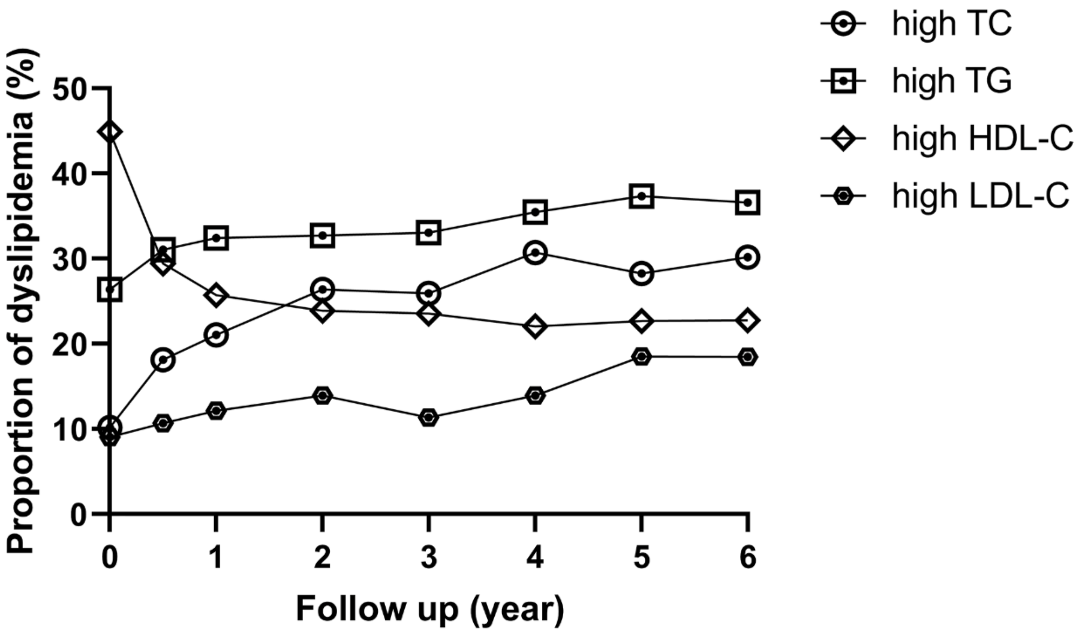

Fig. S1 The proportion of dyslipidemia during the process of ART. ART: antiretroviral therapy; TC, total cholesterol; TG: triglycerides; HDL-C, high-density lipoprotein cholesterol; LDL-C, low-density lipoprotein cholesterol.
